# Adapting two pain assessment tools for young people with cerebral palsy: a multi-stakeholder consensus study

**DOI:** 10.1097/PR9.0000000000001304

**Published:** 2025-06-25

**Authors:** Meredith G. Smith, Rachel J. Gibson, Remo N. Russo, Adrienne R. Harvey

**Affiliations:** a School of Allied Health Science and Practice, University of Adelaide, Adelaide, Australia; b College of Medicine and Public Health, Flinders University, Adelaide, Australia; c Paediatric Rehabilitation Department, Women's and Children's Hospital, Adelaide, Australia; d Neurodisability and Rehabilitation, Murdoch Children's Research Institute, Melbourne, Australia; e Medicine, Dentistry and Health Sciences, University of Melbourne, Melbourne, Australia

**Keywords:** Assessment, Chronic pain, Cerebral palsy, Patient-reported outcome measures

## Abstract

**Introduction:**

Cerebral palsy (CP) is the most common cause of childhood physical disability. Chronic pain is a common comorbidity of CP; however, holistic pain assessment is challenging due to few available self-report tools for people with CP and diverse cognitive, communication, and functional abilities. Previous research has identified 2 tools for potential use in CP (Fear of Pain Questionnaire for Children-Short Form [FOPQ-C-SF] and Modified Brief Pain Inventory [mBPI]), along with potential modifications.

**Objectives:**

To gain consensus on modifications required to the FOPQ-C-SF and mBPI for use with children and young people with CP and chronic pain.

**Methods:**

A 2-round modified electronic Delphi study with people with lived experience (n = 19 and 16) and clinicians (n = 25 and 21) was conducted. Ethics was obtained through the Women's and Children's Health Network Human Research Ethics Committee (2022/HRE00154). A total of 37 modification suggestions to the FOPQ-C-SF and mBPI were rated on a 5-point Likert scale (strongly agree to strongly disagree). Consensus was determined as ≥75% of participants rating the suggestion as strongly agree/agree, or strongly disagree/disagree.

**Results:**

Seven modifications reached consensus for inclusion in the FOPQ-C-SF and 21 for the mBPI. No modifications reached consensus for exclusion for either tool. Modifications primarily focused on refining wording, enhancing visual representation of the tool for improved understanding and reducing the number of response options.

**Conclusion:**

Consensus has been reached on modifications to the FOPQ-C-SF and mBPI for young people with cerebral palsy. Future research will examine the comprehensibility of the adapted tools across the population.

## Abstract 2

Supplemental Digital Content is Available in the Text.

Modifications required to 2 pain assessment tools (Fear of Pain Questionnaire for Children-Short Form and Modified Brief Pain Inventory) to enable people with cerebral palsy and diverse abilities to self-report their pain have been established.

## 1. Introduction

Cerebral palsy (CP) is the leading cause of childhood physical disability worldwide, with a prevalence of approximately 1.6/1000 live births.^22^ Chronic pain, defined as “persistent or recurring pain lasting longer than 3 months,” is more frequently experienced by children and young people with CP than other paediatric populations.^23^ It is well established that chronic pain should be assessed within a biopsychosocial framework, acknowledging the broad impact of chronic pain on a person's life.^19^ Twelve core pain assessment domains have previously been identified for children and young people with CP.^11^ However, few tools that assess pain have evidence of validity for use in CP, or are appropriate for the whole spectrum of abilities seen in CP; for example, communication, cognitive, visual, and motor abilities.^36,39^ Without access to valid and appropriate pain assessment tools, children and young people with CP have limited opportunity to access best practice interventions for chronic pain.^41^

Previous research has identified a need for tools assessing the impact of pain on emotional functioning for children and young people with CP.^17,36,39^ The Pediatrics Initiative on Methods, Measurement and Pain Assessment in Clinical Trials (PedIMMPACT) paediatric chronic pain domains specify emotional functioning, such as anxiety and depression, as a core area of assessment for children with chronic pain.^21^ When core assessment domains were developed specifically for children and young people with CP, this was refined to “impact of pain on emotional functioning” to better distinguish pain-related emotional functioning and general emotional functioning.^11^ Factors contributing to the impact of pain on emotional functioning, such as pain coping, influence pain-related physical disability, anxiety, and depression^7,34^ and are also key targets for pain-related psychological interventions.^8^ Previous work by our team sought to identify which tools focusing on the impact of pain on emotional functioning may be suitable for children and young people with CP.^37^ Briefly, an online survey with clinicians and people with lived experience of CP was conducted to rate the relevance, comprehensiveness, feasibility, and comprehensibility of tools in 2 groups: first, tools that assessed the impact of pain on emotional functioning in a dedicated scale or subscale, and second, multidimensional tools with at least 1 item assessing the impact of pain on emotional functioning. The 2 highest rated tools were the Fear of Pain Questionnaire for Children-Short Form (FOPQ-C-SF) (dedicated scale) and the modified Brief Pain Inventory (mBPI) (multidimensional).^37^ The FOPQ-C-SF was developed by Heathcote et al. (2020)^13^ as a shortened version of the Fear of Pain Questionnaire for Children,^35^ assessing pain-related fear and providing an understanding of the possible impact on pain-related disability.^35^ The Brief Pain Inventory (BPI) was originally developed for adults with cancer pain to measure pain interference, pain intensity, changeable factors, and pain location.^2^ In 2009, Engel et al.^6^ modified the pain interference items of the BPI to be more suitable for children with neuromuscular disorders. The modified version of the BPI was subsequently tested in parents of children with CP as a proxy report measure.^1^ Although both assessment tools have been used in children with CP, until recently there was no investigation of content validity in this population.^36,39^

Our recent study sought to address this gap by investigating the content validity and feasibility of both tools for children and young people with CP and chronic pain.^37^ Briefly, to improve relevance, comprehensibility, and accessibility of the tools across the spectrum of ability, we conducted a qualitative descriptive study with people with lived experience and clinicians. We identified the FOPQ-C-SF and mBPI could be useful in their current form for youth with CP, but only those without cognitive or communication impairment.^37^ The study showed the tools required adaptation of the content to ensure they were relevant and comprehensible for all children and young people with CP. For example, the wording of the mBPI was considered too complex for children or those with cognitive impairment, with phrases like “recreational activities.” Furthermore, the FOPQ-C-SF wording did not reflect the potential reduction in autonomy a person with a disability might experience when they are unable to stop an activity due to external limitations, such as needing assistance to get out of a piece of equipment. Fifty-eight unique modification suggestions were identified from this study; however, a number of the suggestions were conflicting, and the breadth of the recommendation suggestions made implementation difficult. For example, 1 parent identified using a colour scale would improve comprehensibility for their child with CP; however, another parent advised a colour scale would make it less accessible for their child due to visual impairment. Although this was not an unexpected finding given the heterogeneity of the CP population,^28^ further research was recommended to gain consensus on which modifications should be implemented.^37^

Therefore, the primary objective of this current study was to gain consensus among people with lived experience and clinicians on which modifications to the FOPQ-C-SF and mBPI should be implemented to ensure appropriateness for children and young people with CP and chronic pain.

## 2. Methods

### 2.1. Study design

We selected a 2-stage modified electronic Delphi study, conducted between July and September 2023, to facilitate expert consensus on modifications to the tools that had been proposed and discussed in a previous focus group setting, but had not yet reached a conclusion.^11^ A standard Delphi begins with an open-ended question round to generate items, whereas a modified Delphi, as used here, starts with a structured questionnaire based on existing literature.^15^ In this study, the initial survey round was informed by findings from the preceding qualitative study.^37^ The asynchronous nature of the electronic Delphi design also provided greater flexibility, allowing for broader participation from both people with lived experience and clinicians. The study design was guided by COnsensus-based Standards for Measurement Instruments (COSMIN) recommendations.^42^ Our study was codesigned and conducted with a consumer advisory group, including 2 young adults with CP and a parent of an adolescent with CP. The consumer advisory group was involved in defining the study purpose, piloting data collection tools, and reviewing data analysis methods.^14^

Ethics approval was granted through the Women's and Children's Health Network Ethics Committee (2022/HRE00154). All participants provided informed consent, obtained through a consent statement in the survey.

#### 2.1.1. Participants and recruitment

Participants were recruited nationally and internationally to form 2 groups: group 1—clinicians (orthopaedic surgeons, paediatricians, physiotherapists, psychologists, speech pathologists, clinician researchers) and group 2—people with lived experience (children with CP, adults with CP, and parents of children with CP). Participants were eligible for inclusion if they were individuals with a diagnosis of CP (>8 years of age); parents or caregivers of children with CP (child age 2–30 years); and medical, nursing, or allied health clinicians or clinician researchers with >5 years' experience working with children and young adults with CP. Participants were recruited through online advertising and email distribution throughout known networks, including the National Research Group for Pain in Cerebral Palsy, the Australasian Academy for Cerebral Palsy and Developmental Medicine, Novita, the Women's and Children's Hospital Paediatric Rehabilitation Department, and the South Australian Cerebral Palsy Register. All participants were screened with an online demographic survey to ensure they met the inclusion criteria.

#### 2.1.2. Sample size

Although there are no specific guidelines when selecting participant size for a Delphi study, research suggests a minimum of 12 participants is required to reach consensus.^16,26^ For this study, it was important to ensure clinicians had sufficient expertise working with clients with CP (defined as >5 years' experience), as well as ensuring people with lived experience were represented from across the spectrum of mobility and communication ability within CP. To ensure people with lived experience had strong representation in the study, we determined that at least one-third of the study participants should be people with lived experience. We determined a maximum of 60 clinicians/researchers and 30 people with lived experience might be recruited for this study, utilising the methods described. These numbers were based on the membership of the Australasian Academy for Cerebral Palsy and Developmental Medicine (AusACPDM), with 14 members in the national research group for Pain in Cerebral Palsy.

#### 2.1.3. The survey instrument

Survey questions were developed based on the modification suggestions identified by our previous study.^37^ Modification suggestions were reviewed by all authors and the consumer advisory group, and grouped into (1) modifications to be presented in the Delphi study for consensus; (2) modifications that could be implemented without requiring consensus; for example, changes to the administration mode of the tool rather than changes to the tool itself, this included adding alternative methods of access (eg audio); and (3) modifications that were considered inappropriate to take further. Detailed information on the modifications in each group is provided in supplementary material 1, http://links.lww.com/PR9/A325. Modifications that were not taken further included those that would change the construct of interest of the tool. A final list of 38 suggestions were included in the survey instrument (supplementary material 2, http://links.lww.com/PR9/A325). During data analysis, 1 further suggestion was identified as changing the construct of interest and was removed from the analysis (Wording change—FOPQ-C-SF—item 9: “I stop any activity if I start to hurt or my pain becomes worse” to “pain stops me doing things I want to do”). A total of 37 suggestions were included in the analysis (11 relating to the FOPQ-C-SF and 26 relating to the mBPI).

#### 2.1.4. Survey protocol

All potential participants were emailed information regarding the project with a link directly to the electronic Delphi survey. Survey data were collected and managed using REDCap electronic data capture tool, hosted at Murdoch Children's Research Institute.^9,10^ Each round was open for 4 weeks. In round 1, participants were presented with the original FOPQ-C-SF and mBPI and asked to rate each of the suggested changes on a 5-point Likert scale (strongly agree—strongly disagree). An optional “comments” box was included, allowing participants to provide unprompted, open-ended feedback on each individual suggested change. Participants who completed round 1 were subsequently emailed round 2. This round identified modifications that did not reach consensus, and participants were asked to rerate these. Participants were given data on which modifications reached consensus in round 1, along with graphs displaying the percentage responses (strongly agree, agree, neutral, disagree, and strongly disagree) for each modification that did not achieve consensus. The graphs clearly separated the responses from clinicians and people with lived experience, allowing participants to see the differences between the 2 groups. Up to 3 reminder emails were sent for round 2. Modifications that reached consensus for 1 group but not the other (ie, people with lived experience or clinicians) were only re-presented to the group that did not reach consensus.

#### 2.1.5. Data analysis

There are no specific guidelines for determining consensus within a Delphi study.^3,16,27^ For this study, we, therefore, determined consensus was achieved if at least 75% of participants in each group (group 1: clinicians, group 2: people with lived experience) provided consistent ratings:^40^

(1) Consensus: at least 75% of respondents in each group strongly agreed or agreed the modification was appropriate.
(2) Discarded: at least 75% of respondents in each group strongly disagreed or disagreed the modification was appropriate.
(3) Discordant: items for which respondents could not agree. These items were retained and presented again in round 2.

Equal weighting was given across clinicians/researchers and people with lived experience. Optional open-ended responses were coded by 1 author (M.S.) to the predetermined categories of content validity (comprehensibility, comprehensiveness, relevance) and feasibility, as defined by COSMIN.^24^ Open-ended responses were specifically used to contextualise the discordant findings.

## 3. Results

### 3.1. Participant characteristics

Forty-four respondents completed round 1 of the survey (Table 1), including clinicians (n = 25), individuals with CP (n = 9, mean age = 34.48 years), and parents of children with CP (n = 10, mean age of child = 10 years). People with lived experience participants (group 2) were representative of all Gross Motor Function Classification System levels and pain reporting abilities (able to self-report pain, unable to self-report pain, and able to report pain with additional assistance) seen in CP. Thirty-seven respondents (84% retention) completed round 2 of the survey (Table 1), including clinicians (n = 21), individuals with CP (n = 8), and parents of children with CP (n = 8).

**Table 1:** Descriptive statistics of Delphi study participants.

Participant characteristic	% (n)	% (n)
	Round 1 (n = 44)	Round 2 (n = 37)
Clinicians	56.8% (25)	56.8% (21)
Orthopaedic surgeon	4% (1)	0% (0)
Paediatrician	8% (2)	9.5% (2)
Physiotherapist	76% (19)	76.2% (16)
Psychologist	4% (1)	4.8% (1)
Speech pathologist	4% (1)	4.8% (1)
Other	4% (1)	4.8% (1)
People with lived experience	43.2% (19)	43.2% (16)
Young people with CP	20.5% (9)	21.6% (8)
Age range	18–63	18–63
Mean age years (SD)	34.89 (14.04)	35.5 (14.88)
8–18 y	0% (0)	n = 0
18–30 y	55.5% (5)	50% (4)
>30 y	44.5% (4)	50% (4)
Mobility level	n = 9	n = 8
GMFCS I	11.1% (1)	12.5% (1)
GMFCS II	66.7% (6)	75% (6)
GMFCS III	22.2% (2)	12.5% (1)
GMFCS IV	0% (0)	0% (0)
GMFCS V	0% (0)	0% (0)
Communication ability		
Able to report and describe pain without any additional assistance	100% (9)	100% (8)
Able to report and describe pain with the use of a communication device or other method	0% (0)	0% (0)
Unable to report and/or describe pain	0% (0)	0% (0)
Parents of children with CP	22.7% (10)	21.6% (8)
Child age range	3–25	3–25
Mean age of child (SD)	10 (7.5)	10 (7.5)
Mobility level (of child)	n = 10	n = 8
GMFCS I	20% (2)	12.5% (1)
GMFCS II	20% (2)	25% (2)
GMFCS III	20% (2)	12.5% (1)
GMFCS IV	20% (2)	25% (2)
GMFCS V	20% (2)	25% (2)
Communication ability (of child)		
Able to report and describe pain without any additional assistance	70% (7)	62.5% (5)
Able to report and describe pain with the use of a communication device or other method	10% (1)	12.5% (1)
Unable to report and/or describe pain	20% (2)	25% (2)

GMFCS, gross motor function classification system.

### 3.2. Round 1

Of the 37 suggestions presented to participants, 19 achieved consensus (Tables 2 and 3). Four suggestions reached consensus for the FOPQ-C-SF (Table 2), and 15 suggestions for the mBPI (Table 3). No suggestions reached consensus to discard (>75% strongly disagree + disagree responses).

**Table 2:** Round 1 consensus results for fear of pain questionnaire for children-short form modifications.

Suggestion		Experience	Strongly agree % (n)	Agree % (n)	Neutral % (n)	Disagree % (n)	Strongly disagree % (n)	Agree + strongly agree (%)	Disagree + strongly disagree (%)	Consensus rating
Examples FOPQ 1	Examples (item 2: Have a list of examples of what something terrible is, eg, trip over and hurt myself, have to go to hospital)	Lived experience	52.6% (10)	31.6% (6)	15.8% (3)	0% (0)	0% (0)	84.2	0	+
		Clinician	36% (9)	36% (9)	20% (5)	8% (2)	0% (0)	72	8	?
Examples FOPQ 2	Examples (item 9: Have some examples of equipment or therapy as part of “I stop any activity if I start to hurt or my pain becomes worse,” eg, using a standing frame, using a shower chair, using a wheelchair, going to physio)”	Lived experience	26.3% (5)	31.6% (6)	36.8% (7)	5.3% (1)	0% (0)	57.9	5.3	?
		Clinician	28% (7)	36% (9)	16% (4)	16% (4)	4% (1)	64	20	?
Presentation FOPQ 1	Presentation: Make the font & response options larger and have bigger spacing between	Lived experience	52.6% (10)	31.6% (6)	10.5% (2)	5.3% (1)	0% (0)	84.2	5.3	+
		Clinician	40% (10)	52% (13)	8% (2)	0% (0)	0% (0)	92	0	+
Presentation FOPQ 2	Presentation: Have a version with only 1 question/item per page (including a picture, along with the response scale)	Lived experience	31.6% (6)	36.8% (7)	21.1% (4)	10.5% (2)	0% (0)	68.4	10.5	?
		Clinician	24% (6)	36% (9)	28% (7)	8% (2)	4% (1)	60	12	?
Proxy report FOPQ	Parent report: Do not include a parent report option for the Fear of Pain Questionnaire as it is too individual/personal and cannot be answered accurately by a parent	Lived experience	21.1% (4)	21.1% (4)	26.3% (5)	26.3% (5)	5.3% (1)	42.2	31.6	?
		Clinician	4% (1)	16% (4)	36% (9)	40% (10)	4% (1)	20	44	?
Response option FOPQ 1	Responses: Add colour background to the response options, eg, changes from green to yellow to red as the score increases, eg, changes from lighter shade to darker shade of the same colour as the score increases	Lived experience	36.8% (7)	31.6% (6)	21.1% (4)	10.5% (2)	0% (0)	68.4	10.5	?
		Clinician	12% (3)	24% (6)	48% (12)	16% (4)	0% (0)	36	16	?
Response option FOPQ 2	Responses: Add visual symbols as well as numbers, eg, 2 thumbs up, 1 thumb up, thumbs in the middle, 1 thumb down, 2 thumbs down	Lived experience	36.8% (7)	47.4% (9)	10.5% (2)	5.3% (1)	0% (0)	84.2	5.3	+
		Clinician	24% (6)	40% (10)	20% (5)	16% (4)	0% (0)	64	16	?
Visuals FOPQ 1	Visuals: Add pictures to each item, eg, “pain makes me feel scared” has a picture of someone looking scared	Lived experience	36.8% (7)	42.1% (8)	21.1% (4)	0% (0)	0% (0)	78.9	0	+
		Clinician	28% (7)	40% (10)	24% (6)	8% (2)	0% (0)	68	8	?
Wording FOPQ 1	Wording change (item 1: Remove the word “normal”: changes to “I can't do all the things other people do because it's so easy to hurt my body”)	Lived experience	78.9% (15)	15.8% (3)	0% (0)	5.3% (1)	0% (0)	94.7	5.3	+
		Clinician	76% (19)	24% (6)	0% (0)	0% (0)	0% (0)	100	0	+
Wording FOPQ 2	Wording change (item 4: Change “I cancel plans” to “I don't want to do things” or “I don't want to go to things”)	Lived experience	57.9% (11)	26.3% (5)	10.5% (2)	0% (0)	5.3% (1)	84.2	5.3	+
		Clinician	52% (13)	28% (7)	8% (2)	12% (3)	0% (0)	80	12	+
Wording FOPQ 3	Wording change (items 4,7,8,10: Change words which assume autonomy to “I wish I could” or “I want to,” eg, Change “I do not go to school because it makes my pain worse” to “I don't want to go to school because it makes my pain worse”)	Lived experience	52.6% (10)	36.8% (7)	5.3% (1)	5.3% (1)	0% (0)	89.4	5.3	+
		Clinician	48% (12)	40% (10)	8% (2)	4% (1)	0% (0)	88	4	+

Consensus rating: +, consensus reached for inclusion; ?, no consensus reached; —, consensus reached for exclusion.

**Table 3:** Round 1 consensus results for modified brief pain inventory modifications.

Suggestion		Experience	Strongly agree % (n)	Agree % (n)	Neutral % (n)	Disagree % (n)	Strongly disagree % (n)	Agree + strongly agree (%)	Disagree + strongly disagree (%)	Consensus rating
Addition mBPI 1	Wording addition (item 4: school/work to school/work/day activities)	Lived experience	42.1% (8)	36.8% (7)	15.8% (3)	5.3% (1)	0% (0)	78.9	5.3	+
		Clinician	60% (15)	32% (8)	0% (0)	8% (2)	0% (0)	92	8	+
Addition mBPI 2	Item addition: Add a blank space to record a favourite activity and then rate how pain gets in the way of that	Lived experience	36.8% (7)	42.1% (8)	15.8% (3)	0% (0)	5.3% (1)	78.9	5.3	+
		Clinician	56% (14)	40% (10)	4% (1)	0% (0)	0% (0)	96	0	+
Addition mBPI 3	Addition: Add a comments section at the bottom to answer “is there a time when you don't have pain?” or “is there something you do that makes your pain better?”	Lived experience	47.4% (9)	36.8% (7)	15.8% (3)	0% (0)	0% (0)	84.2	0	+
		Clinician	48% (12)	40% (10)	8% (2)	4% (1)	0% (0)	88	4	+
Examples mBPI 1	Examples: Add examples/descriptions to each item, eg, recreational activities = playing games with friends, going to the movies, swimming, listening to music	Lived experience	57.9% (11)	36.8% (7)	5.3% (1)	0% (0)	0% (0)	94.7	0	+
		Clinician	48% (12)	44% (11)	4% (1)	4% (1)	0% (0)	92	4	+
Examples mBPI 2	Examples: Add optional examples of equipment/assistive technology, eg, general activity (item 1—include examples such as getting around in my walker/wheelchair/crutches)	Lived experience	47.4% (9)	42.1% (8)	10.5% (2)	0% (0)	0% (0)	89.5	0	+
		Clinician	36% (9)	44% (11)	16% (4)	4% (1)	0% (0)	80	4	+
Presentation mBPI 1	Presentation: Make the font larger and have bigger spacing	Lived experience	52.6% (10)	36.8% (7)	5.3% (1)	5.3% (1)	0% (0)	89.4	5.3	+
		Clinician	44% (11)	44% (11)	12% (3)	0% (0)	0% (0)	88	0	+
Presentation mBPI 2	Presentation: Alternate row colours so it is easier to see	Lived experience	26.3% (5)	36.8% (7)	21.1% (4)	15.8% (3)	0% (0)	63.1	15.8	?
		Clinician	36% (9)	28% (7)	24% (6)	12% (3)	0% (0)	64	12	?
Presentation mBPI 3	Presentation: Have a version with only 1 question/item per page (including a picture, along with the response scale)	Lived experience	26.3% (5)	42.1% (8)	21.1% (4)	10.5% (2)	0% (0)	68.4	10.5	?
		Clinician	20% (5)	32% (8)	36% (9)	8% (2)	4% (1)	52	12	?
Proxy report mBPI	Parent report: A parent report option is appropriate for the modified brief pain inventory questions about how pain gets in the way of life	Lived experience	47.4% (9)	36.8% (7)	15.8% (3)	0% (0)	0% (0)	84.2	0	+
		Clinician	44% (11)	44% (11)	8% (2)	4% (1)	0% (0)	88	4	+
Response option mBPI 1	Responses: Have a version with less response options (ie, reducing from 0 to 10 to 0–5)	Lived experience	52.6% (10)	15.8% (3)	21.1% (4)	10.5% (2)	0% (0)	68.4	10.5	?
		Clinician	32% (8)	44% (11)	12% (3)	12% (3)	0% (0)	76	12	+
Response option mBPI 2	Responses: Add colour background to the response options, eg, changes from green to yellow to red as the score increases, eg, changes from lighter shade to darker shade of the same colour as the score increases	Lived experience	36.8% (7)	36.8% (7)	5.3% (1)	21.1% (4)	0% (0)	73.6	21.1	?
		Clinician	12% (3)	12% (3)	52% (13)	20% (5)	4% (1)	24	24	?
Response option mBPI 3	Responses: Add visual symbols as well as numbers eg, happy face, okay face, unhappy face	Lived experience	36.8% (7)	47.4% (9)	10.5% (2)	5.3% (1)	0% (0)	84.2	5.3	+
		Clinician	28% (7)	36% (9)	20% (5)	16% (4)	0% (0)	64	16	?
Response option mBPI 4	Responses: Add descriptions along the response option scale, eg, on a 0–5 scale: 0 = not at all, 2 = a fair bit, 5 = a lot	Lived experience	36.8% (7)	57.9% (11)	5.3% (1)	0% (0)	0% (0)	94.7	0	+
		Clinician	36% (9)	48% (12)	12% (3)	4% (1)	0% (0)	84	4	+
Response option mBPI 5	Responses: Add “I don't know” or “I don't understand” as a response option This is a prompt for people who use communication devices to be able to communicate that they need more explanation to answer the question	Lived experience	73.7% (14)	26.3% (5)	0% (0)	0% (0)	0% (0)	100	0	+
		Clinician	48% (12)	40% (10)	4% (1)	8% (2)	0% (0)	88	8	+
Visuals mBPI 1	Visuals: Add pictures to each item, eg, taking care of daily needs (item 6 has pictures of showering, getting dressed eg, recreational activities (item 5 has pictures of outdoor activities, swimming and sport))	Lived experience	52.6% (10)	36.8% (7)	5.3% (1)	5.3% (1)	0% (0)	89.4	5.3	+
		Clinician	36% (9)	60% (15)	4% (1)	0% (0)	0% (0)	96	0	+
Visuals mBPI 2	Visuals: Include pictures of people using mobility aids and communication devices, eg, recreational activities (item 5—has pictures of people with playing games but may be using a mobility aid, eg, communication with others (item 9 has pictures of a person using a high or low tech communication device))	Lived experience	47.4% (9)	36.8% (7)	15.8% (3)	0% (0)	0% (0)	84.2	0	+
		Clinician	48% (12)	52% (13)	0% (0)	0% (0)	0% (0)	100	0	+
Wording mBPI 1	Wording change: interferes changes to gets in the way	Lived experience	42.1% (8)	42.1% (8)	15.8% (3)	0% (0)	0% (0)	84.2	0	+
		Clinician	28% (7)	48% (12)	8% (2)	16% (4)	0% (0)	76	16	+
Wording mBPI 10	Wording change (item 11: social activities to spending time with friends and family)	Lived experience	31.6% (6)	42.1% (8)	21.1% (4)	5.3% (1)	0% (0)	73.7	5.3	?
		Clinician	44% (11)	32% (8)	16% (4)	8% (2)	0% (0)	76	8	+
Wording mBPI 2	Wording change (item 1: general activity to getting around)	Lived experience	36.8% (7)	21.1% (4)	36.8% (7)	5.3% (1)	0% (0)	57.9	5.3	?
		Clinician	36% (9)	32% (8)	8% (2)	20% (5)	4% (1)	68	24	?
Wording mBPI 3	Wording change (item 3: mood to feelings)	Lived experience	31.6% (6)	26.3% (5)	26.3% (5)	15.8% (3)	0% (0)	57.9	15.8	?
		Clinician	52% (13)	20% (5)	4% (1)	24% (6)	0% (0)	72	24	?
Wording mBPI 4	Wording change (item 5: recreational activity to play or things I do for fun)	Lived experience	42.1% (8)	47.4% (9)	10.5% (2)	0% (0)	0% (0)	89.5	0	+
		Clinician	48% (12)	32% (8)	0% (0)	20% (5)	0% (0)	80	20	+
Wording mBPI 5	Wording change (item 6: taking care of daily needs to looking after myself)	Lived experience	47.4% (9)	21.1% (4)	26.3% (5)	5.3% (1)	0% (0)	68.5	5.3	?
		Clinician	36% (9)	40% (10)	16% (4)	8% (2)	0% (0)	76	8	+
Wording mBPI 6	Wording change (item 7: learning new information or skills to learning new things or concentrating)	Lived experience	31.6% (6)	52.6% (10)	15.8% (3)	0% (0)	0% (0)	84.2	0	+
		Clinician	36% (9)	44% (11)	4% (1)	16% (4)	0% (0)	80	16	+
Wording mBPI 7	Wording change (item 8: relations with others to friendships or getting along with others)	Lived experience	57.9% (11)	26.3% (5)	10.5% (2)	5.3% (1)	0% (0)	84.2	5.3	+
		Clinician	56% (14)	40% (10)	0% (0)	4% (1)	0% (0)	96	4	+
Wording mBPI 8	Wording change (item 9: communication with others to tell people what I want to tell them)	Lived experience	31.6% (6)	21.1% (4)	26.3% (5)	21.1% (4)	0% (0)	52.7	21.1	?
		Clinician	36% (9)	24% (6)	8% (2)	28% (7)	4% (1)	60	32	?
Wording mBPI 9	Wording change (item 10: enjoyment of life to having fun)	Lived experience	31.6% (6)	36.8% (7)	31.6% (6)	0% (0)	0% (0)	68.4	0	?
		Clinician	44% (11)	24% (6)	20% (5)	12% (3)	0% (0)	68	12	?

Consensus rating: +, consensus reached for inclusion; ?, no consensus reached; —, consensus reached for exclusion.

### 3.3. Round 2

After round 2, a further 9 changes reached consensus for inclusion (total 28/37): 3 changes to the FOPQ-C-SF (Table 4) and 6 to the mBPI (Table 5). No changes reached consensus to discard, and 9 changes remained discordant. Tables 6 and 7 detail the changes that reached consensus and those that were discordant.

**Table 4:** Round 2 consensus results for fear of pain questionnaire for children-short form modifications.

Suggestion		Experience	Strongly agree % (n)	Agree % (n)	Neutral % (n)	Disagree % (n)	Strongly disagree % (n)	Agree + strongly agree (%)	Disagree + strongly disagree (%)	Consensus rating
Examples FOPQ 1	Examples (item 2: Have a list of examples of what something terrible is, eg, trip over and hurt myself, have to go to hospital)	Clinician	38.1% (8)	38.1% (8)	4.8% (1)	14.3% (3)	4.8% (1)	76.2	19.1	+
Examples FOPQ 2	Examples (item 9: Have some examples of equipment or therapy as part of “I stop any activity if I start to hurt or my pain becomes worse,” eg, using a standing frame, using a shower chair, using a wheelchair, going to physio)	Lived experience	18.8% (3)	50% (8)	31.2% (5)	0% (0)	0% (0)	68.8	0	?
		Clinician	23.8% (5)	28.6% (6)	38.1% (8)	4.8% (1)	4.8% (1)	52.4	9.6	?
Presentation FOPQ 2	Presentation: Have a version with only 1 question/item per page (including a picture, along with the response scale)	Lived experience	6.2% (1)	62.5% (10)	18.8% (3)	6.2% (1)	6.2% (1)	68.7	12.4	?
		Clinician	0% (0)	57.1% (12)	23.8% (5)	14.3% (3)	4.8% (1)	57.1	19.1	?
Proxy report FOPQ	Parent report: Do not include a parent report option for the Fear of Pain Questionnaire as it is too individual/personal and cannot be answered accurately by a parent	Lived experience	12.5% (2)	6.2% (1)	37.5% (6)	31.2% (5)	12.5% (2)	18.7	43.7	?
		Clinician	0% (0)	14.3% (3)	19% (4)	52.4% (11)	14.3% (3)	14.3	66.7	?
Response option FOPQ 1	Responses: Add colour background to the response options, eg, changes from green to yellow to red as the score increases, eg, changes from lighter shade to darker shade of the same colour as the score increases	Lived experience	12.5% (2)	50% (8)	25% (4)	12.5% (2)	0% (0)	62.5	12.5	?
		Clinician	9.5% (2)	57.1% (12)	28.6% (6)	4.8% (1)	0% (0)	66.6	4.8	?
Response option FOPQ 2	Responses: Add visual symbols as well as numbers, eg, thumbs up/thumbs down	Clinician	9.5% (2)	66.7% (14)	23.8% (5)	0% (0)	0% (0)	76.2	0	+
Visuals FOPQ 1	Visuals: Add pictures to each item, eg, “pain makes me feel scared” has a picture of someone looking scared	Clinician	9.5% (2)	76.2% (16)	14.3% (3)	0% (0)	0% (0)	85.7	0	+

Consensus rating: +, consensus reached for inclusion; ?, no consensus reached; —, consensus reached for exclusion.

**Table 5:** Round 2 consensus results for modified brief pain inventory modifications.

Suggestion		Experience	Strongly agree % (n)	Agree % (n)	Neutral % (n)	Disagree % (n)	Strongly disagree % (n)	Agree + strongly agree (%)	Disagree + strongly disagree (%)	Consensus rating
Presentation mBPI 2	Presentation: Alternate row colours so it is easier to see	Lived experience	25% (4)	56.2% (9)	6.2% (1)	6.2% (1)	6.2% (1)	81.2	12.4	+
		Clinician	38.1% (8)	52.4% (11)	4.8% (1)	4.8% (1)	0% (0)	90.5	4.8	+
Presentation mBPI 3	Presentation: Have a version with only 1 question/item per page (including a picture, along with the response scale)	Lived experience	6.2% (1)	50% (8)	31.2% (5)	6.2% (1)	6.2% (1)	56.2	12.4	?
		Clinician	0% (0)	57.1% (12)	28.6% (6)	9.5% (2)	4.8% (1)	57.1	14.3	?
Response option mBPI 1	Responses: Have a version with less response options (ie, reducing from 0 to 10 to 0–5)	Lived experience	37.5% (6)	37.5% (6)	18.8% (3)	0% (0)	6.2% (1)	75	6.2	+
Response option mBPI 2	Responses: Add colour background to the response options, eg, changes from green to yellow to red as the score increases, eg, changes from lighter shade to darker shade of the same colour as the score increases	Lived experience	12.5% (2)	37.5% (6)	37.5% (6)	6.2% (1)	6.2% (1)	50	12.4	?
		Clinician	14.3% (3)	47.6% (10)	33.3% (7)	4.8% (1)	0% (0)	61.9	4.8	?
Response option mBPI 3	Responses: Add visual symbols as well as numbers, eg, happy face, okay face, unhappy face	Clinician	4.8% (1)	85.7% (18)	4.8% (1)	0% (0)	4.8% (1)	90.5	4.8	+
Wording mBPI 10	Wording change (item 11: social activities to spending time with friends and family)	Lived experience	43.8% (7)	43.8% (7)	6.2% (1)	0% (0)	6.2% (1)	87.6	6.2	+
Wording mBPI 2	Wording change (item 1: general activity to getting around)	Lived experience	25% (4)	37.5% (6)	25% (4)	0% (0)	12.5% (2)	62.5	12.5	?
		Clinician	28.6% (6)	52.4% (11)	9.5% (2)	9.5% (2)	0% (0)	81	9.5	+
Wording mBPI 3	Wording change (item 3: mood to feelings)	Lived experience	56.2% (9)	25% (4)	12.5% (2)	6.2% (1)	0% (0)	81.2	6.2	+
		Clinician	23.8% (5)	42.9% (9)	14.3% (3)	19% (4)	0% (0)	66.7	19	?
Wording mBPI 5	Wording change (item 6: taking care of daily needs to looking after myself)	Lived experience	62.5% (10)	25% (4)	6.2% (1)	0% (0)	6.2% (1)	87.5	6.2	+
Wording mBPI 8	Wording change (item 9: communication with others to tell people what I want to tell them)	Lived experience	31.2% (5)	31.2% (5)	25% (4)	6.2% (1)	6.2% (1)	62.4	12.4	?
		Clinician	38.1% (8)	38.1% (8)	4.8% (1)	9.5% (2)	9.5% (2)	76.2	19	+
Wording mBPI 9	Wording change (item 10: enjoyment of life to having fun)	Lived experience	43.8% (7)	37.5% (6)	6.2% (1)	0% (0)	12.5% (2)	81.3	12.5	+
		Clinician	23.8% (5)	57.1% (12)	14.3% (3)	0% (0)	4.8% (1)	80.9	4.8	+

Consensus rating: +, consensus reached for inclusion; ?, no consensus reached; —, consensus reached for exclusion.

**Table 6:** Results from round 2: consensus changes to modified brief pain inventory and fear of pain questionnaire for children-short form.

Suggestions which reached consensus to include
>75% clinicians and >75% people with lived experience agreed or strongly agreed with the suggested change

Change type	Change
Fear of Pain Questionnaire	
Presentation	Make the font & response options larger and have bigger spacing between
Wording change (item 1)	Remove the word “normal” Changes to: “I can't do all the things other people do because it's so easy to hurt my body”
Wording change (items 4, 7, 8, 10)	Change words that assume autonomy to I wish I could or I want to, eg, Change “I do not go to school because it makes my pain worse” to “I don't want to go to school because it makes my pain worse”
Wording change (item 4)	Change “I cancel plans” to “I don't want to do things” or “I don't want to go to things”
Examples (item 2)	Have a list of examples of what something terrible is, eg, trip over and hurt myself, have to go to hospital
Response option	Add visual symbols as well as numbers, eg, 2 thumbs up, 1 thumb up, thumbs in the middle, 1 thumb down, 2 thumbs down
Visuals	Add pictures to each item, eg, feelings of pain are scary for me has a picture of someone looking scared
Modified Brief Pain Inventory	
Addition	Add a comments section at the bottom to answer “is there a time when you don't have pain?” or “is there something you do that makes your pain better?”
Examples	Add examples/descriptions to each item, eg, recreational activities = *playing games with friends, going to the movies, swimming, listening to music*
Examples	Add optional examples of equipment/assistive technology, eg, *general activity (item 1)—Include examples such as getting around in my walker/wheelchair/crutches*
Item addition	Add a blank space to record a favourite activity and then rate how pain gets in the way of that
Presentation	Make the font larger and have bigger spacing
Presentation	Alternate row colours so it is easier to see
Parent report	A parent report option is appropriate for the modified brief pain inventory questions about how pain gets in the way of life
Responses	Add descriptions along the response option scale, eg, *on a 0–5 scale: 0 = not at all, 2 = a fair bit, 5 = a lot*
Responses	Have a version with less response options (ie, reducing from 0–10 to 0–5)
Responses	Add “I don't know” as a response option
Responses	Add visual symbols as well as numbers, eg, happy face, okay face, unhappy face
Visuals	Add pictures to each item, eg, *taking care of daily needs (item 6) has pictures of showering, getting dressed* eg, *recreational activities (item 5) has pictures of outdoor activities, swimming and sport*
Visuals	Include pictures of people using mobility aids and communication devices, eg, *recreational activities (item 5)—has pictures of people with playing games but may be using a mobility aid* eg, *communication with others (item 9) has pictures of a person using a high or low tech communication device*
Wording change	“Interferes” changes to “gets in the way”
Wording addition (item 4)	Change “school/work” to “school/work/day activities”
Wording change (item 5)	“Recreational activity” to “play” or “things I do for fun”
Wording change (item 6)	“Taking care of daily needs” to “looking after myself”
Wording change (item 7)	“Learning new information or skills” to “learning new things” or “concentrating”
Wording change (item 8)	“Relations with others” to “friendships” or “getting along with others”
Wording change (item 10)	“Enjoyment of life” to “having fun”
Wording change (item 11)	“Social activities” to “spending time with friends and family”

**Table 7:** Results from round 2: discordant suggested changes to modified brief pain inventory and fear of pain questionnaire for children-short form.

Response category definitions
Relevance: Suggestions relating to the relevance or meaning of the items for people with cerebral palsy
Comprehensiveness: Suggestions to ensure the full scope of the construct is covered, as is relevant to people with cerebral palsy
Comprehensibility: Suggestions related to an individual's ability to understand the tool—including but not limited to understanding the items, wording, purpose of the tool, and response options
Feasibility: Suggestions related to the use of the tool in clinical practice and research by clinicians and people with lived experience

Suggestions which were discordant					
Fear of pain questionnaire					
Change type	Change	Clinicians (agree/strongly agree %) No. of participants who included an open-ended comment n =	People with lived experience (agree/strongly agree %) No. of participants who included an open-ended comment n =	Open-ended response category	Summary
Examples (item 9)	Have some examples of equipment or therapy as part of “I stop any activity if I start to hurt or my pain becomes worse,” eg, using a standing frame, using a shower chair, using a wheelchair, going to physio	52% n = 2	69% n = 1	Relevance Comprehensiveness	Implies the activities are only related to therapy Could bias the answers of those with less physical disability (ie, those who don't use equipment)
Presentation	Have a version with only 1 question/item per page (including a picture, along with the response scale)	57% n = 3	69% n = 1	Feasibility	Challenging in clinical settings due to multiple pieces of paper, would be suitable as an electronic version
Proxy report	Parent report: Do not include a parent report option for the Fear of Pain Questionnaire as it is too individual/personal and cannot be answered accurately by a parent	Agree/strongly agree: 14% Disagree/strongly disagree: (67%) n = 8	Agree/strongly agree 19% Disagree/strongly disagree: (43%) n = 2	Feasibility Relevance	Useful to know the parent's own beliefs around pain (rather than the parent's perspective of the child's beliefs about pain). Sometimes the child won't be able to report at all, and in this case, parent information could be useful
Response option	Add colour background to the response options, eg, changes from green to yellow to red as the score increases e.g. changes from lighter shade to darker shade of the same colour as the score increases	67% n = 4	63% n = 2	Feasibility Comprehensibility	Increased visual complexity for those with visual impairment Colours could influence the responses as they can have an emotional overlay

Modified brief pain inventory					
Change type	Change	Clinicians No. of participants who included an open-ended comment n =	People with lived experience No. of participants who included an open-ended comment n =	Open-ended response category	Summary
Presentation	Have a version with only 1 question/item per page (including a picture, along with the response scale)	57% n = 2	56% n = 0	Feasibility	Challenging in clinical settings due to multiple pieces of paper
Responses	Add colour background to the response options, eg, changes from green to yellow to red as the score increases, eg, changes from lighter shade to darker shade of the same colour as the score increases	62% n = 3	50% n = 3	Feasibility Comprehensibility	Increased visual complexity for those with visual impairment Colours could influence the responses as they can have an emotional overlay
Wording change (item 1)	“General activity” to “getting around”	81% n = 4	63% n = 1	Comprehensiveness Relevance	General activity is more than just “getting around.” It should also encompass other activities that are not mobility dependent
Wording change (item 3)	“Mood” to “feelings”	67% n = 5	81% n = 2	Comprehensibility	Mood and feelings can be interpreted differently. Feelings is too broad, mood more clearly relates to “low mood” (ie, pain, anxiety, depression)
Wording change (item 9)	“Communication with others” to “tell people what I want to tell them”	76% n = 6	62% n = 3	Comprehensiveness	Communication is more than just telling, it also needs to include listening, understanding, and being understood

### 3.4. Agreement between groups

In round 1, 4 suggested changes reached consensus for people with lived experience but not for clinicians (Table 2). All 4 of these changes reached consensus with clinicians in round 2. Three suggested changes in round 1 reached consensus for clinicians but not people with lived experience (Table 4); again all of these changes reached consensus with people with lived experience in round 2. At the end of round 2, only 3 suggested changes, all related to the mBPI, had reached consensus with 1 group only (Table 7).

## 4. Discussion

This study highlighted the importance of modifying assessment tools to improve the validity and feasibility of the tools for use in young people with CP and chronic pain. Clinicians and people with lived experience gained consensus on modifications to the FOPQ-C-SF and the mBPI to improve appropriateness and accessibility for young people with CP. These modifications are now suitable for implementation and pilot testing in children and young people with CP and varying communication and cognitive abilities.

The modifications to be implemented include wording adjustment, improvement of the visual presentation of the tool, use of visuals to aid understanding, and a reduction in the number of response options. The desire to include visuals and/or symbols is unsurprising, with Rinde et al.^32^ reporting good success in engaging children with CP, complex communication needs, and reduced cognitive abilities in discussion about their pain when using visual aids. Similarly, reducing the number of response options has been shown to improve the reliability of self-reported pain intensity in typically developing 3 and 4 year olds.^5^ Adding examples of equipment or assistive technology to mBPI items, for instance mobility (item 12 mBPI) = getting around in my walker/wheelchair/crutches, reached consensus. This is important with research conducted by Dubois et al.^4^ reporting standing frame use was more frequently associated with pain than any other care-related acts experienced by children with CP. The wording changes in the FOPQ-C-SF also recognise the reduced autonomy children and young people with disability may have as a result of mobility, cognition, or communication limitations.^33^

Feasibility and content validity are both key in recommending a tool for use in clinical practice.^31^ Feasibility is crucial, as it determines the uptake of a tool by people with lived experience and clinicians in clinical practice.^25^ Content validity is considered the most important measurement property, as it determines whether a tool measures what it intends to measure, including relevance to the population of interest, relevance to the construct of interest, comprehensiveness, and comprehensibility for the target population.^31^ Our previous qualitative study investigated the content validity and feasibility of the original mBPI and FOPQ-C-SF, generating suggestions for improving these tools for children and young people with CP. Subsequently, this current study focused on selecting the most important modifications to enhance relevance, comprehensiveness, comprehensibility, and feasibility for this population, without changing the construct of interest, ready for further testing. Unfortunately, we did not have any children with CP (<18 years) willing to participate in the Delphi process, although they were eligible to be included. Although it is possible this may have skewed the modifications that did reach consensus, the diversity of adults with CP and parents of children with CP included was likely sufficient to overcome this. Although this is a limitation of this study, we do not consider it a significant limitation in the overall adaptation of the tools as our previous research has thoroughly investigated both the relevance and comprehensiveness of the modified versions of the FOPQ-C-SF and mBPI through qualitative interviews with children with CP. We are also cognisant that comprehensibility requires further testing across a broader range of children with CP and cognitive and communication impairment; it was not possible to include the full breadth of cognitive and communication impairment in this study or our previous qualitative study due to the complexity of the original versions of the tools.

Two wording suggestions for the mBPI reached consensus with clinicians but not people with lived experience. Whilst clinicians suggested changing “general activity” to “getting around,” which essentially combined general activity and mobility into 1 item, a number of participants provided open-ended responses indicating general activity is “more than just moving around” (Table 7). We, therefore, suggest the wording of “general activity” be further investigated with children and young people with CP. A further suggestion for the mBPI, changing “mood” to “feelings” reached consensus for people with lived experience but not clinicians. Open-ended response comments from clinicians suggested the term “feelings” is much broader than “mood,” and “feelings” are not necessarily linked with anxiety, depression, and pain in the same way as “mood” (Table 7). We recommend this also needs further investigation.

One suggestion for this study was to have a version of each assessment tool listing 1 question or item per page. Although the 3 lived experience participants representing children with complex communication needs all agreed or strongly agreed with this suggestion, it did not reach consensus in either group. Clinician feedback indicated potential difficulty of implementing an assessment with multiple pages in clinical practice; however, this feedback also suggested use of a digital device may make this feasible.^37^ Despite this suggestion not reaching consensus, given the importance of this modification for Augmentative and Alternative Communication users, we recommend it is further considered and tested in children with complex communication needs.

A number of the modifications to the tools related to comprehensibility, including simplifying wording, using visuals to assist understanding and simplifying response categories. Most paediatric patient-reported outcome measures (PROMs) advise self-report questionnaires are suitable for children aged 8 years and above.^20^ This age recommendation is based on cognitive ability. However, as 1 in 2 children with CP have an intellectual disability,^28^ setting the standard for self-reporting at a cognitive age of 8 years potentially excludes a large proportion of the population from ever accessing self-report measures. Given the higher prevalence of chronic pain in the CP population,^23^ consideration needs to be given as to how best to adapt PROMs to be more accessible to children with cognitive abilities below 8 years of age.^18^ This is especially important considering the presence of both motor and cognitive disabilities, limited communication skills and frequent care interventions place these individuals at a high risk of experiencing repeated induced pain.^4^ Emmott et al.^5^ have been considering how to improve access to self-reported pain intensity measures in typically developing children as young as 3 years of age. Despite the challenges in eliciting self-report in young children, some degree of self-report is still preferred given the reduced accuracy of proxy report.^5,30,36^ This can be performed in conjunction with parent proxy report where the accuracy of the child's self-report is unclear.

The need for holistic and accessible pain assessment for children with developmental disabilities like CP is increasingly recognised.^29^ Further, ensuring all 12 core pain domains for CP^11^ are assessed requires clinicians to think more broadly than just pain intensity.^38^ The FOPQ-C-SF and mBPI can both be used to assess the impact of pain on emotional functioning. The mBPI can be used as a multidimensional tool to contribute to assessment of this domain, and the FOPQ-C-SF provides assessment of pain-related fear and activity avoidance.^35^ Routine assessment of the impact of pain on emotional functioning may improve access to currently underutilised best practice multidisciplinary interventions for children and young people with CP.^41^

Our study had a number of strengths. Participants in the study had close to equal representation of clinicians and people with lived experience, and lived experience participants were representative of the spectrum of abilities seen in the CP population. We also used a modified 2 stage Delphi design, which likely improved participation in round 2. A response rate of 84% was achieved in round 2, which is well above the recommended minimum response rate of 70% for subsequent rounds of a Delphi study.^12^ It is possible the summary of round 1 results influenced participants' responses in round 2. However, this is standard Delphi procedure.^40^ The results for the lived experience and clinician groups were presented separately to acknowledge group effects and minimise the influence of clinicians on people with lived experience.^27^ A study limitation was that although a range of clinician disciplines were represented, most participants were physiotherapists. Although this may have potentially skewed which modifications reached consensus, this was managed through assigning equal weighting to both people with lived experience and clinicians. Furthermore, as it is highly likely physiotherapists will be one of the larger discipline groups using the modified tools in clinical practice, we do not consider this a significant limitation. Finally, the survey included an optional open-ended comment box for each suggested change, which not all participants completed. As a result, the comments may not fully represent the entire sample. The open-ended comments were also only coded by 1 author. However, since these comments were used only to provide possible explanations and context for changes that did not reach consensus, this is not considered a significant limitation.

## 5. Conclusion

Consensus has been gained on modifications to the FOPQ-C-SF and mBPI to improve accessibility and self-report opportunity for all children and young people with CP. Future research will involve implementing these modifications and then pilot testing the tools across the communication, cognitive, and motor ability spectrum of children and young people with CP. This will allow for these assessment tools to be used in clinical practice to increase access to best practice chronic pain interventions for children and young people with CP.

## Disclosures

No funding or sponsorship was received for this study. The authors have stated that they had no interests that might be perceived as posing a conflict or bias.

## Appendix A. Supplemental digital content

Supplemental digital content associated with this article can be found online at http://links.lww.com/PR9/A325.

## Supplementary Material

SUPPLEMENTARY MATERIAL
