# Supplementary material for: Adapting two pain assessment tools for young people with cerebral palsy: a multi-stakeholder consensus study
Source: Pain Rep. 2025 Jun 25;10(4):e1304. doi: 10.1097/PR9.0000000000001304 (PMC12200239; doi:10.1097/PR9.0000000000001304)
Supplement: SUPPLEMENTARY MATERIAL [file painreports-10-e1304-s001.pdf]

Supplementary material 1: Suggested changes from qualitative descriptive study grouped into those to be presented in round 1 of the Delphi, those that do not need Delphi consensus and those not to be presented

Each suggested change from the qualitative study is displayed in the table below. The categories the change was coded to are indicated, along with the number of participants who suggested that change; and the category of participant (people with lived experience or clinicians).

Definitions of the coding categories are provided below:

Category	Category description
**Accessibility**	Suggestions for how people with disability can access the tool to prioritise self-report
**Comprehensibility**	Comments related to an individual's ability to understand the tool - including but not limited to understanding the items, wording, purpose of the tool and response options
**Comprehensiveness**	Suggestions to ensure the full scope of the construct is covered, as is relevant to people with cerebral palsy. The focus here is on clarifying existing items, not adding new items
**Feasibility**	Suggestions related to the use of the tool in clinical practice and research by clinicians and people with lived experience
**Presentation**	How the assessment tool is displayed or presented
**Relevance**	Suggestions relating to the relevance or meaning of the tool for people with cerebral palsy
**Suggested changes**	All suggested practical changes/modifications to the tools

Further information on the study which generated the suggested changes can be found here:

Smith MG, Gibson RJ, Russo RN, Karanicolas S, Harvey AR. Examining tools for assessing the impact of chronic pain on emotional functioning in children and young people with cerebral palsy: stakeholder preference and recommendations for modification. Qual Life Res. 2024 Aug;33(8):2247-2259. doi: 10.1007/s11136-024-03693-1. Epub 2024 May 25. PMID: 38795198; PMCID: PMC11286630.

**During data analysis, this suggestion was identified as changing the construct of interest and was removed from the analysis*

Suggested changes and modifications to the tools to take to the Delphi survey	Accessibility	Comprehensibility	Comprehensiveness	Feasibility	Presentation	Relevance	Clinicians (/12)	Lived experience (/18)	No. participants (/30)
**1. Add visual symbols for each item**	X	X			X	X	X	X	10
a) <u>mBPI</u>							X	X	4
General activity- include pictures of moving around using different mobility aids					X		X		1
Recreational activities- pictures of outdoor activities and sports		X				X		X	2
**2. Changes to wording**	X	X	X	X	X	X	X	X	29
a) Change words which assume autonomy to 'I wish I could' or 'I want to'	X	X	X		X	X	X	X	10
b) <u>FOPQ-C</u>		X	X	X	X	X	X	X	20
Add other anxiety symptoms to 'pain causes my heart to beat fast'		X				X		X	5
Add standardised examples or descriptions for each item				X			X	X	2
Change 'I cancel plans' to 'I don't want to do things' or 'I don't want to go to things'		X				X	X		1
*Change 'I stop doing things when I'm in pain' to 'pain stops me doing things I want to do'**		X					X		1
Clarify what 'something terrible' is (e.g. adding examples such as 'trip over and hurt myself', 'have to go to hospital')		X			X	X	X	X	5
Remove the word 'normal' from item 1 and simplify the wording		X		X		X	X	X	9

Suggested changes and modifications to the tools to take to the Delphi survey	Accessibility	Comprehensibility	Comprehensiveness	Feasibility	Presentation	Relevance	Clinicians (/12)	Lived experience (/18)	No. participants (/30)
c) mBPI	X	X	X	X	X	X	X	X	28
Add standardised examples or descriptions to each item		X	X	X	X	X	X	X	18
Change 'communication with others' to 'tell people what I'd like to tell them''		X		X	X		X	X	6
Change 'general activity' to 'getting around'		X				X	X	X	10
Change 'interferes' to 'gets in the way of' or 'stops me'	X	X			X		X	X	9
Change 'learning new information or skills' to 'learning new things' or 'concentration'		X					X		4
Change 'mood' to 'feelings'	X	X					X	X	8
Change 'recreational activity' to 'play' or 'things I do for fun'		X				X	X	X	14
Change 'relations with others' to 'friendships' or 'playing with my friends' or 'getting along with others' or 'spending time with friends and family'		X				X	X	X	12
Change 'taking care of daily needs' to 'looking after myself'		X				X	X	X	2
Consider leaving out 'enjoyment of life' for a child or changing to 'having fun'		X			X	X	X	X	6
**3. Items that could be added**			X	X	X	X	X	X	16
a) FOPQ-C			X	X	X	X	X	X	10
Add 'fear of therapy, equipment, medical intervention, doctor'			X	X		X	X	X	9

Suggested changes and modifications to the tools to take to the Delphi survey	**Accessibility**	**Comprehensibility**	**Comprehensiveness**	**Feasibility**	**Presentation**	**Relevance**	**Clinicians** (/12)	**Lived experience** (/18)	No. participants (/30)
b) mBPI			X			X	X	X	6
Add a blank option for favourite activity			X			X	X	X	2
Add a prompt or comment about how pain interferes with ability to use equipment and assistive technology			X					X	1
Add 'is there a time when you are pain free?' comment			X				X	X	2
**4. Presentation**	X				X			X	3
a) Make font & boxes larger with bigger spacing	X				X			X	2
b) Alternate row colours (grey/white)					X			X	1
c) Present questions one at a time	X	X		X	X			X	4
**5. Proxy report versions** Include a parent report option for mBPI but not FOPQ - the FOPQ is too individual/personal and cannot be answered accurately by a parent	X			X			X	X	10
**6. Response options**	X	X	X	X	X	X	X	X	29
a) Add a colour background to the response option symbols (i.e. green to red)	X	X			X		X	X	8
b) Add 'I don't know' and 'not applicable' as response options	X	X		X			X	X	2
c) Add visual symbols as well as numbers		X			X		X	X	11
d) Add words as well as numbers and visuals		X			X		X	X	5

Suggested changes and modifications to the tools to take to the Delphi survey	Accessibility	Comprehensibility	Comprehensiveness	Feasibility	Presentation	Relevance	Clinicians (/12)	Lived experience (/18)	No. participants (/30)
e) Change the number of response options to less than 10 (0-5 and/or 0-3)	X	X			X		X	X	5

**Suggested changes that don't need to go to the Delphi**<br><br>*(We will include these as part of the modified tools, but don't need to get consensus on them as they are not wording or scale changes)*	**Accessibility**	**Comprehensibility**	**Comprehensiveness**	**Feasibility**	**Presentation**	**Relevance**	**Clinicians**	**Lived experience**	**No. participants (/30)**
**1. Administration instructions**	X	X		X	X		X	X	21
a) Clinicians must feedback the results to the client or family				X	X		X	X	3
b) Clinicians to explain what the assessment is used for				X			X	X	4
c) Complete prior to an appointment				X			X	X	10
d) Complete with a support person or caregiver				X				X	7
e) Have an option for audio	X	X		X	X		X	X	3
f) Set of quick instructions for users	x	x		x			x		3
g) FOPQ - Change order of questions					X		X		5
h) Ensure it is switch accessible		X			X			X	2
**2. Have different versions of the tool for people with different needs**	X	X		X	X		X	X	4
a) Electronic and printable versions of the tool	X			X	X		X		1
**3.** Presentation changes									
a) Add a comments section - FOPQ					X		X	X	2

Suggested changes that are not going through to the Delphi survey	Accessibility	Comprehensibility	Comprehensivenes	Feasibility	Presentation	Relevance	Clinicians	Lived experience	No. participants	Reason for removal
Could use PODD symbols as visuals					X			X	1	Not all children use PODDs, it would be more inclusive to use pictures/visuals generally
Include meal times as one of the visuals for 'taking care of daily needs' (mBPI)							X		1	Only suggested by one clinician, will see what children say on pilot testing (this may be included anyway). Meal times suggests only eating
Every person with CP should be given the FOPQ as they might not know that their pain is related to fear				X				X	1	Practically, it is not possible or necessarily appropriate to give to everyone. Can be used at clinician discretion. Only suggested by one person
Add wording options for children younger than school age				X			X		1	Aiming to find wording that is accessible to younger children already
Change 'I hurt' to 'When I hurt' (FOPQ)							X		1	Semantics, only suggested by one person
Change wording from negative to positive (FOPQ)				X	X		X		1	Changing to positive wording changes the construct of the tool (fear of pain)
Change wording from ‘feelings of pain are scary for me’ to 'I'm scared to do XXXX because of my pain' (FOPQ)			X				X		2	Only referenced once, changes the meaning of the item
Combine ‘put things off’ and ‘avoid making plans’ (FOPQ)					X	X		X	1	Changing number of items will change structural validity of the tool (has existing evidence for structural validity)
Specify not mobilising or moving because of pain (FOPQ)			X				X		1	Can include this as a standardised example/description
Comment option for ‘time of day’ pain interferes most (mBPI)			X					X	1	Only one reference, also difficult when assessing chronic pain to identify a time of day
Add something about 'how I feel about pain in the future'(mBPI)			X				X		1	Only one reference and suggested by a clinician

Consider changing strongly agree and disagree to a frequency scale (e.g. a lot, a little) (FOPQ)						X	X		1	Changes the scale too much – will need to test the entire scale/structural validity again. Only one suggestion

Supplementary material 2: Delphi Round 1

Rating Modifications Survey - Chronic Pain Assessment In Cerebral Palsy

Welcome to this survey to improve pain assessment in people with cerebral palsy.

The top rated assessment tools were:

Modified Brief Pain Inventory

Fear of Pain Questionnaire - Child

Both of these questionnaires have been reviewed by people with lived experience (children and adults with cerebral palsy and parents of children with cerebral palsy) and clinicians. We will now present the suggested changes to you for your feedback.

Please rate each suggestion from strongly agree - strongly disagree.

This should take approximately 10 minutes to complete

Please note - there are some things we cannot modify without affecting the validity of the tool. These include:

	**Can modify**	**Can't modify**
**Wording**	Making questions simpler/easier to understand	Wording which changes the meaning of the question
**Presentation**	Adding pictures/symbols, changing the layout	
**Examples**	Examples given with the questions to be more relevant to people with cerebral palsy	
**Response options**	Adding an option to answer 0-5 rather than 0-10	Changing a scale from strongly disagree/agree to a frequency (all the time, none of the time)

Tool 1:

The modified Brief Pain Inventory (mBPI) is a multidimensional tool for chronic pain that includes the domain 'impact on emotional functioning'

Sample of the original Modified Brief Pain Inventory:

(scroll down for questions)

clicking on the image below will open the sample in a new tab if needed

---

Questions start here:

Please rate each suggestion from 'strongly agree' - 'strongly disagree'

1 Visuals: Add pictures to each item
e.g. 'taking care of daily needs' (item 6) has pictures of showering, getting dressed

e.g. 'recreational activities' (item 5) has pictures of outdoor activities, swimming and sport

◯ Strongly agree
◯ Agree
◯ Neutral
◯ Disagree
◯ Strongly disagree

Comments (optional)
(Optional)

2 Visuals: Include pictures of people using mobility aids and communication devices
e.g. 'recreational activities' (item 5) - has pictures of people with playing games but may be using a mobility aid

e.g. 'communication with others' (item 9) has pictures of a person using a high or low tech communication device

◯ Strongly agree
◯ Agree
◯ Neutral
◯ Disagree
◯ Strongly disagree

Comments (optional)
(Optional)

3 Examples: Add examples/descriptions to each item
e.g. 'recreational activities' = playing games with friends, going to the movies, swimming, listening to music

◯ Strongly agree
◯ Agree
◯ Neutral
◯ Disagree
◯ Strongly disagree

Comments (optional)
(Optional)

4 Examples: Add optional examples of equipment/assistive technology
e.g. 'general activity' (item 1) - include examples such as 'getting around in my walker/wheelchair/crutches'

◯ Strongly agree
◯ Agree
◯ Neutral
◯ Disagree
◯ Strongly disagree

Comments (optional)
(Optional)

5 Responses: Have a version with less response options (i.e. reducing from 0-10 to 0-5)

◯ Strongly agree
◯ Agree
◯ Neutral
◯ Disagree
◯ Strongly disagree

Comments (optional)
(Optional)

6 Responses: Add colour background to the response options
e.g. changes from green to yellow to red as the score increases

e.g. changes from lighter shade to darker shade of the same colour as the score increases

◯ Strongly agree
◯ Agree
◯ Neutral
◯ Disagree
◯ Strongly disagree

Comments (optional)
(Optional)

7 Responses: Add visual symbols as well as numbers
e.g. happy face, okay face, unhappy face

◯ Strongly agree
◯ Agree
◯ Neutral
◯ Disagree
◯ Strongly disagree

Comments (optional)
(Optional)

8 Responses: Add descriptions along the response option scale
e.g. on a 0-5 scale: 0 = 'not at all', 2 = 'a fair bit', 5 = 'a lot'

◯ Strongly agree
◯ Agree
◯ Neutral
◯ Disagree
◯ Strongly disagree

Comments (optional)
(Optional)

9 Responses: Add 'I don't know' or 'I don't understand' as a response option

This is a prompt for people who use communication devices to be able to communicate that they need more explanation to answer the question

◯ Strongly agree
◯ Agree
◯ Neutral
◯ Disagree
◯ Strongly disagree

Comments (optional)
(Optional)

10 Wording change: 'interferes' changes to 'gets in the way'

◯ Strongly agree
◯ Agree
◯ Neutral
◯ Disagree
◯ Strongly disagree

Comments (optional)
(Optional)

11 Wording change (item 1): 'general activity' to 'getting around'

◯ Strongly agree
◯ Agree
◯ Neutral
◯ Disagree
◯ Strongly disagree

Comments (optional)
(Optional)

12 Wording change (item 3): 'mood' to 'feelings'

◯ Strongly agree
◯ Agree
◯ Neutral
◯ Disagree
◯ Strongly disagree

Comments (optional)
(Optional)

13 Wording addition (item 4): 'school/work' to 'school/work/day activities'

◯ Strongly agree
◯ Agree
◯ Neutral
◯ Disagree
◯ Strongly disagree

Comments (optional)
(Optional)

14 Wording change (item 5): 'recreational activity' to 'play' or 'things I do for fun'

◯ Strongly agree
◯ Agree
◯ Neutral
◯ Disagree
◯ Strongly disagree

Comments (optional)
(Optional)

15 Wording change (item 6): 'taking care of daily needs' to 'looking after myself'

◯ Strongly agree
◯ Agree
◯ Neutral
◯ Disagree
◯ Strongly disagree

Comments (optional)
(Optional)

16 Wording change (item 7): 'learning new information or skills' to 'learning new things' or 'concentrating'

◯ Strongly agree
◯ Agree
◯ Neutral
◯ Disagree
◯ Strongly disagree

Comments (optional)
(Optional)

17 Wording change (item 8): 'relations with others' to 'friendships' or 'getting along with others'

◯ Strongly agree
◯ Agree
◯ Neutral
◯ Disagree
◯ Strongly disagree

Comments (optional)
(Optional)

18 Wording change (item 9): 'communication with others' to 'tell people what I want to tell them'

◯ Strongly agree
◯ Agree
◯ Neutral
◯ Disagree
◯ Strongly disagree

Comments (optional)
(Optional)

19 Wording change (item 10): 'enjoyment of life' to 'having fun'

◯ Strongly agree
◯ Agree
◯ Neutral
◯ Disagree
◯ Strongly disagree

Comments (optional)
(Optional)

20 Wording change (item 11): 'social activities' to 'spending time with friends and family'

◯ Strongly agree
◯ Agree
◯ Neutral
◯ Disagree
◯ Strongly disagree

Comments (optional)
(Optional)

21 Item addition: add a blank space to record a favourite activity and then rate how pain gets in the way of that

◯ Strongly agree
◯ Agree
◯ Neutral
◯ Disagree
◯ Strongly disagree

Comments (optional)
(Optional)

22 Addition: Add a comments section at the bottom to answer 'is there a time when you don't have pain?' or 'is there something you do that makes your pain better?'

◯ Strongly agree
◯ Agree
◯ Neutral
◯ Disagree
◯ Strongly disagree

Comments (optional)
(Optional)

23 Presentation: Make the font larger and have bigger spacing

◯ Strongly agree
◯ Agree
◯ Neutral
◯ Disagree
◯ Strongly disagree

Comments (optional)
(Optional)

24 Presentation: Alternate row colours so it is easier to see

◯ Strongly agree
◯ Agree
◯ Neutral
◯ Disagree
◯ Strongly disagree

Comments (optional)
(Optional)

25 Presentation: Have a version with only one question/item per page (including a picture), along with the response scale

◯ Strongly agree
◯ Agree
◯ Neutral
◯ Disagree
◯ Strongly disagree

Comments (optional)
(Optional)

26 Parent report: A parent report option is appropriate for the modified brief pain inventory questions about how pain gets in the way of life

◯ Strongly agree
◯ Agree
◯ Neutral
◯ Disagree
◯ Strongly disagree

Comments (optional)
(Optional)

Any additional comments?

1/2 complete

.

Tool 2:

The Fear of Pain Questionnaire is a pain coping tool for measuring the impact of chronic pain on emotional functioning

Sample of the original Fear of Pain Questionnaire for Children - short form:

(scroll down for questions)

clicking on the image below will open the sample in a new tab if needed

Questions start here:

27 Visuals: Add pictures to each item
e.g. 'feelings of pain are scary for me' has a picture of someone looking scared

◯ Strongly agree
◯ Agree
◯ Neutral
◯ Disagree
◯ Strongly disagree

Comments (optional)
(Optional)

28 Wording change (item 1): Remove the word 'normal'

Changes to "I can't do all the things other people do because it's so easy to hurt my body'

◯ Strongly agree
◯ Agree
◯ Neutral
◯ Disagree
◯ Strongly disagree

Comments (optional)
(Optional)

29 Examples (item 2): Have a list of examples of what 'something terrible' is

e.g. 'trip over and hurt myself', 'have to go to hospital'

◯ Strongly agree
◯ Agree
◯ Neutral
◯ Disagree
◯ Strongly disagree

Comments (optional)
(Optional)

30 Wording change (item 4): Change "I cancel plans" to "I don't want to do things" or "I don't want to go to things"

◯ Strongly agree
◯ Agree
◯ Neutral
◯ Disagree
◯ Strongly disagree

Comments (optional)
(Optional)

31 Wording change (items 4,7,8,10): Change words which assume autonomy to 'I wish I could' or 'I want to'
e.g. Change 'I do not go to school because it makes my pain worse' to 'I don't want to go to school because it makes my pain worse'

◯ Strongly agree
◯ Agree
◯ Neutral
◯ Disagree
◯ Strongly disagree

Comments (optional)
(Optional)

32 Wording change (item 9): "I stop any activity if I start to hurt or my pain becomes worse' to "pain stops me doing things I want to do"

◯ Strongly agree
◯ Agree
◯ Neutral
◯ Disagree
◯ Strongly disagree

Comments (optional)
(Optional)

33 Examples (item 9): Have some examples of equipment or therapy as part of ''I stop any activity if I start to hurt or my pain becomes worse"

e.g. using a standing frame, using a shower chair, using a wheelchair, going to physio

◯ Strongly agree
◯ Agree
◯ Neutral
◯ Disagree
◯ Strongly disagree

Comments (optional)
(Optional)

34 Responses: Add colour background to the response options
e.g. changes from green to yellow to red as the score increases

e.g. changes from lighter shade to darker shade of the same colour as the score increases

◯ Strongly agree
◯ Agree
◯ Neutral
◯ Disagree
◯ Strongly disagree

Comments (optional)
(Optional)

35 Responses: Add visual symbols as well as numbers
e.g. two thumbs up, one thumb up, thumbs in the middle, one thumb down, two thumbs down

◯ Strongly agree
◯ Agree
◯ Neutral
◯ Disagree
◯ Strongly disagree

Comments (optional)
(Optional)

36 Presentation: Make the font & response options larger and have bigger spacing between

◯ Strongly agree
◯ Agree
◯ Neutral
◯ Disagree
◯ Strongly disagree

Comments (optional)
(Optional)

37 Presentation: Have a version with only one question/item per page (including a picture), along with the response scale

◯ Strongly agree
◯ Agree
◯ Neutral
◯ Disagree
◯ Strongly disagree

Comments (optional)
(Optional)

38 Parent report: Do not include a parent report option for the Fear of Pain Questionnaire as it is too individual/personal and cannot be answered accurately by a parent

◯ Strongly agree
◯ Agree
◯ Neutral
◯ Disagree
◯ Strongly disagree

Comments (optional)
(Optional)

Any additional comments?

2/2 complete

.

Delphi Round 2 Chronic Pain Assessment In Cerebral Palsy

Welcome to the final round of the chronic pain assessment in cerebral palsy survey!

We have reached consensus on a number of suggested changes to the questionnaires

- (list here if you would like to view).

There are 18 suggested changes that did not reach consensus, and we are asking you to look at these again. The results from the previous survey are shown for your information and we would like you to rate these again.

Your answer does not need to be the same as the previous round.

This should take 5 minutes to complete.

Thank you!

Suggested changes to Tool 1:

The modified Brief Pain Inventory (mBPI) is a multidimensional tool for chronic pain that includes the domain 'impact on emotional functioning'

Sample of the original Modified Brief Pain Inventory:

(scroll down for questions)

clicking on the link above will open the sample in a new tab if needed

Questions start here:

Responses: Have a version with less response options (i.e. reducing from 0-10 to 0-5)

Results from previous round:

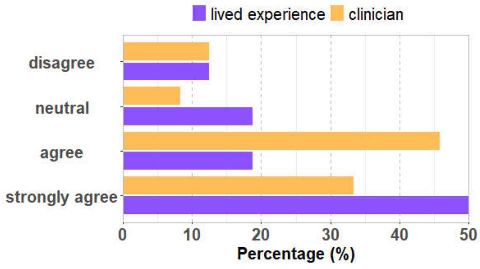

- ◯ Strongly agree
- ◯ Agree
- ◯ Neutral
- ◯ Disagree
- ◯ Strongly disagree

Comments (optional)
(Optional)

Responses: Add colour background to the response options
e.g. changes from green to yellow to red as the score increases

e.g. changes from lighter shade to darker shade of the same colour as the score increases

Results from previous round:

◯ Strongly agree
◯ Agree
◯ Neutral
◯ Disagree
◯ Strongly disagree

Comments (optional)
(Optional)

Responses: Add visual symbols as well as numbers
e.g. happy face, okay face, unhappy face

Results from previous round:

◯ Strongly agree
◯ Agree
◯ Neutral
◯ Disagree
◯ Strongly disagree

Comments (optional)
(Optional)

Wording change (item 1): 'general activity' to 'getting around'

Results from previous round:

◯ Strongly agree
◯ Agree
◯ Neutral
◯ Disagree
◯ Strongly disagree

Comments (optional)
(Optional)

Wording change (item 3): 'mood' to 'feelings'

Results from previous round:

◯ Strongly agree
◯ Agree
◯ Neutral
◯ Disagree
◯ Strongly disagree

Comments (optional)
(Optional)

Wording change (item 6): 'taking care of daily needs' to 'looking after myself'

Results from previous round:

◯ Strongly agree
◯ Agree
◯ Neutral
◯ Disagree
◯ Strongly disagree

Comments (optional)
(Optional)

Wording change (item 9): 'communication with others' to 'tell people what I want to tell them'

Results from previous round:

◯ Strongly agree
◯ Agree
◯ Neutral
◯ Disagree
◯ Strongly disagree

Comments (optional)
(Optional)

Wording change (item 10): 'enjoyment of life' to 'having fun'

Results from previous round:

◯ Strongly agree
◯ Agree
◯ Neutral
◯ Disagree
◯ Strongly disagree

Comments (optional)
(Optional)

Wording change (item 11): 'social activities' to 'spending time with friends and family'

Results from previous round:

◯ Strongly agree
◯ Agree
◯ Neutral
◯ Disagree
◯ Strongly disagree

Comments (optional)
(Optional)

Presentation: Alternate row colours so it is easier to see

Results from previous round:

◯ Strongly agree
◯ Agree
◯ Neutral
◯ Disagree
◯ Strongly disagree

Comments (optional)
(Optional)

Presentation: Have a version with only one question/item per page (including a picture), along with the response scale

Results from previous round:

◯ Strongly agree
◯ Agree
◯ Neutral
◯ Disagree
◯ Strongly disagree

Comments (optional)
(Optional)

Any additional comments?

1/2 complete

.

Tool 2:

The Fear of Pain Questionnaire is an pain coping tool for measuring the impact of chronic pain on emotional functioning

Sample of the original Fear of Pain Questionnaire for Children - short form:

(scroll down for questions)

clicking on the link above will open the sample in a new tab if needed

Questions start here:

Visuals: Add pictures to each item
e.g. 'feelings of pain are scary for me' has a picture of someone looking scared

Results from previous round:

◯ Strongly agree
◯ Agree
◯ Neutral
◯ Disagree
◯ Strongly disagree

Comments (optional)
(Optional)

Wording change (item 9): "I stop any activity if I start to hurt or my pain becomes worse' to "pain stops me doing things I want to do"

Results from previous round:

◯ Strongly agree
◯ Agree
◯ Neutral
◯ Disagree
◯ Strongly disagree

Comments (optional)
(Optional)

Examples (item 2): Have a list of examples of what 'something terrible' is

e.g. 'trip over and hurt myself', 'have to go to hospital'

◯ Strongly agree
◯ Agree
◯ Neutral
◯ Disagree
◯ Strongly disagree

Comments (optional)
(Optional)

Examples (item 9): Have some examples of equipment or therapy as part of ''I stop any activity if I start to hurt or my pain becomes worse"

e.g. using a standing frame, using a shower chair, using a wheelchair, going to physio

◯ Strongly agree
◯ Agree
◯ Neutral
◯ Disagree
◯ Strongly disagree

Comments (optional)
(Optional)

Responses: Add colour background to the response options
e.g. changes from green to yellow to red as the score increases

e.g. changes from lighter shade to darker shade of the same colour as the score increases

Results from previous round:

◯ Strongly agree
◯ Agree
◯ Neutral
◯ Disagree
◯ Strongly disagree

Comments (optional)
(Optional)

Responses: Add visual symbols as well as numbers
e.g. two thumbs up, one thumb up, thumbs in the middle, one thumb down, two thumbs down

Results from previous round:

◯ Strongly agree
◯ Agree
◯ Neutral
◯ Disagree
◯ Strongly disagree

Comments (optional)
(Optional)

Presentation: Have a version with only one question/item per page (including a picture), along with the response scale

Results from previous round:

◯ Strongly agree
◯ Agree
◯ Neutral
◯ Disagree
◯ Strongly disagree

Comments (optional)
(Optional)

Parent report: Do not include a parent report option for the Fear of Pain Questionnaire as it is too individual/personal and cannot be answered accurately by a parent

Results from previous round:

◯ Strongly agree
◯ Agree
◯ Neutral
◯ Disagree
◯ Strongly disagree

Comments (optional)
(Optional)

Any additional comments?

2/2 complete

.
